# Systematic Identification and Molecular Characteristics of Long Noncoding RNAs in Pig Tissues

**DOI:** 10.1155/2017/6152582

**Published:** 2017-09-14

**Authors:** Yalan Yang, Rong Zhou, Shiyun Zhu, Xunbi Li, Hua Li, Hui Yu, Kui Li

**Affiliations:** ^1^College of Life Science, Foshan University, Foshan, Guangdong 528231, China; ^2^Department of Pig Genomic Design and Breeding, Agricultural Genome Institute at Shenzhen, Chinese Academy of Agricultural Sciences, Shenzhen, Guangdong 518124, China

## Abstract

Long noncoding RNAs (lncRNAs) are non-protein-coding RNAs that are involved in a variety of biological processes. The pig is an important farm animal and an ideal biomedical model. In this study, we performed a genome-wide scan for lncRNAs in multiple tissue types from pigs. A total of 118 million paired-end 90 nt clean reads were obtained via strand-specific RNA sequencing, 80.4% of which were aligned to the pig reference genome. We developed a stringent bioinformatics pipeline to identify 2,139 high-quality multiexonic lncRNAs. The characteristic analysis revealed that the novel lncRNAs showed relatively shorter transcript length, fewer exons, and lower expression levels in comparison with protein-coding genes (PCGs). The guanine-cytosine (GC) content of the protein-coding exons and introns was significantly higher than that of the lncRNAs. Moreover, the single nucleotide polymorphism (SNP) density of lncRNAs was significantly higher than that of PCGs. Conservation analysis revealed that most lncRNAs were evolutionarily conserved among pigs, humans, and mice, such as CUFF.253988.1, which shares homology with human long noncoding RNA* MALAT1*. The findings of our study significantly increase the number of known lncRNAs in pigs.

## 1. Introduction

The discovery of new classes of regulatory noncoding RNAs (ncRNAs), which constitute a majority of transcriptional products, challenges the central dogma of biology [1]. Noncoding RNAs are generally classified as small RNAs (fewer than 200 nt), a group that includes microRNAs, piwi-interacting RNAs, and small nucleolar RNAs, or as long noncoding RNAs (lncRNAs, more than 200 nt). lncRNAs share many similar features with mRNA, such as multiexonic structures, 5′ caps, and polyadenylation, but the former group lacks coding potential [2]. The last decade has witnessed the identification of thousands of lncRNAs in humans [3], animals [4], and plants [5]. Although the functions of most lncRNAs remain unknown, a large body of evidence has revealed that lncRNAs generally exhibit tissue-specific or developmental stage-specific expression patterns [6, 7] and are involved in a broad range of functions, including chromatin modification [8], imprinting [9], transcription [10], splicing [11], posttranscriptional processing [12], and translation [13]. It may be possible to predict the functions of lncRNAs by analyzing their expression signatures and examining the genomic context of lncRNAs relative to that of protein-coding genes with known functions [14]. Moreover, although lncRNAs are generally evolutionarily conserved to a lesser degree than are protein-coding genes, thousands of lncRNAs are conserved across species [15–19].

The domestic pig* (Sus scrofa)* is a major animal protein source for human and has significant advantages over other biomedical models [20, 21]. Some lncRNAs are known to be associated with complex and economically relevant traits in pigs [22–24]. TncRNA, a porcine lncRNA isolated from long SAGE libraries, may perform complex and critical functions in pig fetal development [24]. Further studies found relationships between lncRNAs and pig embryo before implantation [25], skeletal muscle development [22, 23, 26], and obesity [27]. However, the sequences of pig lncRNAs are difficult to infer from other mammalian genomes because of the low sequence conservation of lncRNAs, which causes them to be mistaken for transcriptional noise [1]. Therefore, systematic identification of pig lncRNAs and analysis of their characteristics are necessary to provide a foundation for further studies of the biological functions of noncoding RNAs in this important model species.

To gain insight into the characteristics of* Sus scrofa *lncRNAs, total RNA, excluding rRNA, was isolated and pooled from different tissues at different developmental stages and sequenced using strand-specific RNA sequencing. We identified 2,139 novel* Sus scrofa* lncRNAs in this study. The transcripts were assembled, after which a computational pipeline was developed to screen novel lncRNAs. The sequences and structural features of putative lncRNAs were also analyzed. This study provides a catalog of porcine lncRNAs to serve as a foundation for further studies on the functions and evolutionary history of noncoding RNAs in mammals.

## 2. Materials and Methods

### 2.1. Sample Collection

Tissue was harvested from Landrace, Tongcheng, and Wuzhishan pigs during different developmental stages. The collected samples included tissues from the* longissimus dorsi*, heart, spleen, lung, liver, kidney, stomach, small intestine, and leg muscle. Samples were frozen in liquid nitrogen and stored at −80°C until RNA isolation. Slaughter and embryonic manipulations were carried out in accordance with the protocols of the Chinese Academy of Agricultural Sciences and the Institutional Animal Care and Use Committee.

### 2.2. Illumina Sequencing

Total RNA was isolated from tissue samples from fetuses (33, 40, 50, 55, 60, 65, 70, 75, 80, 85, 90, 95, 100, and 105 dpc), piglets (postnatal days 0 and 10), and adult pigs using Trizol reagent (Invitrogen, Carlsbad, CA, USA) according to the manufacturer's protocols and pooled. RNA was treated with DNase I (Invitrogen, Carlsbad, CA, USA) to remove genomic DNA. Ribosomal RNA was removed from the total RNA using Epicentre's Ribo-Zero rRNA. RNA quality was assessed with an Agilent 2100 Bioanalyzer system (Agilent Technologies, CA, USA). A mixed library was constructed by mixing equal quantities of each RNA sample. A strand-specific library for 90 bp paired-end sequencing was prepared according to the dUTP second strand method [28]. The library was sequenced on an Illumina Genome Analyzer II platform. The transcriptome data generated have been deposited in NCBI Sequence Read Archive with accession number SRP112393 (http://www.ncbi.nlm.nih.gov/Traces/sra/).

### 2.3. Transcript Assembly

After trimming the adaptor sequences, removing low-quality reads, and filtering ribosomal RNA using custom scripts, processed reads were mapped to the reference genome (*Sus scrofa *10.2) by TopHat2 (v2.1.0) with default parameters [29]. Mapped reads were assembled into transcripts using Cufflinks (v1.3.0) [30] with the assistance of known annotations downloaded from the Ensembl database (release 78). The assembled transcripts were used to identify lncRNAs in pigs.

### 2.4. Pipeline for Discovery and Identification of lncRNAs

**Figure 1:**
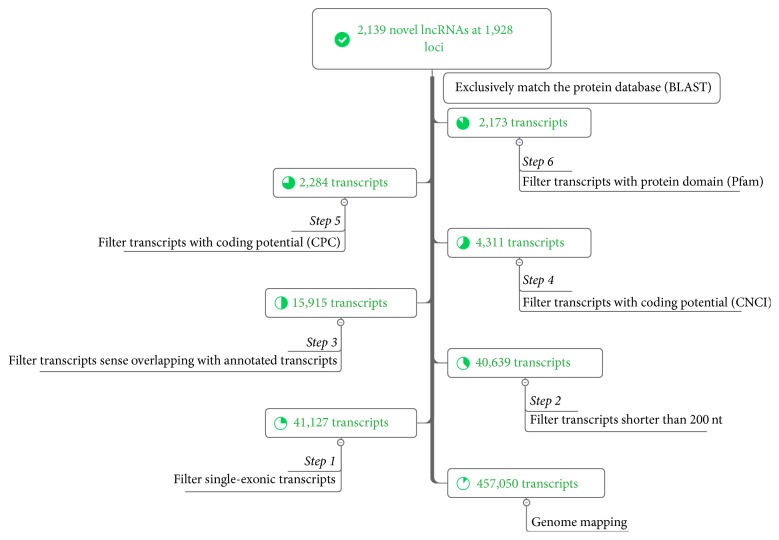
Pipeline for predicting novel lncRNAs.

We used a computational method to identify pig lncRNAs. In our pipeline (Figure 1), seven steps were utilized to screen the assembled sequences for putative lncRNAs. First, single-exon transcripts were filtered to remove unreliable transcripts owing to the complexity of transcriptional reconstruction. Next, long transcripts (>200 nt) and those that did not overlap with known genes were retained for further analysis. Subsequently, two programs, Coding Potential Calculator (CPC, version 0.9-r2) [31] and Coding-Non-Coding Index (CNCI, version 2) [32], were used to distinguish protein-coding genes from noncoding genes. CPC discriminates coding transcripts from noncoding transcripts based on biological features, including homology to known protein sequences and the presence and quality of ORFs. CNCI classifies protein-coding and noncoding sequences by profiling adjoining nucleotide triplets. Only transcripts with both CPC and CNCI scores less than 0 were regarded as noncoding potentiality. All remaining transcripts whose corresponding translated protein sequences had a known protein-coding domain in the Pfam database (version 30.0) were also removed. Finally, transcripts with similarity to known proteins in the UniRef90 protein database were removed using blastx (BLAST 2.2.26+) with an *E*-value cutoff of 10^−5^. The remaining transcripts were designated as putative lncRNAs. Moreover, the coding potential of putative lncRNAs was further assessed and validated by Coding Potential Assessment Tool (CPAT, v1.2.2) software [33].

### 2.5. Characterization of Putative lncRNAs

The exon numbers, lengths, and expression levels of the putative lncRNAs were compared to those of protein-coding transcripts. The expression levels of protein-coding genes and lncRNAs were measured as fragments per kilobase of exon per million fragments mapped (FPKM). The chromosome coordinates of four regions (exons, introns, 1000 bp upstream of transcript, and 1000 bp downstream of transcript) were obtained for protein-coding transcripts and lncRNAs according to annotation files. Random genome regions were selected using the random function in BEDtools [34] with a windows size of 1000 bp. SNP density was calculated based on the* Sus scrofa* dbSNP Build 147, which was downloaded from the NCBI (https://www.ncbi.nlm.nih.gov/). GC content and SNP density were calculated using BEDtools [34].

### 2.6. Conversation of lncRNAs

We used the* Sus scrofa* 10.2 genome assembly as the reference genome. PhyloFit from PHAST package [35] was used to compute phylogenetic model for conserved and nonconserved regions among pig, human, and mouse, and then this model and HMM transition parameters were set for phastCons [35] to compute the conservation scores of lncRNAs and protein-coding transcripts. The conservation status of pig lncRNAs across species was analyzed using the LiftOver tool based on the chain files of pairwise alignments of susScr3ToMm10 and susScr3ToHg38 produced by the UCSC comparative genomics pipeline [36]. lncRNAs were considered as conserved lncRNAs when 50% of its nucleotides uniquely intersected with an alignment in the chain file (coverage ≥ 50%). lncRNAs were denoted as pig-specific lncRNAs if they did not overlap with any alignments in either chain file. In addition, we identified transcript-level conserved lncRNAs according to methods of our previous study [23]. We aligned the identified pig lncRNAs with lncRNAs in human and mouse deposited in NONCODE database [37] by blastn using parameters “–word_size 6 -evalue 0.01 -strand plus”.

### 2.7. Real-Time Quantitative PCR (RT-qPCR)

The tissue expression profile of CUFF.253988.1 was evaluated by RT-qPCR in Yorkshire pigs at the age of 180 days. Total RNA was reverse-transcribed into cDNA using RevertAid First Strand cDNA Synthesis Kit (Thermo, Waltham, MA, USA) according to the manufacturer's protocols. RT-qPCR primers of CUFF.253988.1 were as follows: forward primer: 5′-TCAACTTTAATTTGTGGTGGTGC-3′; reverse primers: 5′-CTCGCTCTTGAATTTATCGTCC-3′. Porcine* GAPDH* gene was selected as reference controls (forward primer: 5′-AGGGCATCCTGGGCTACACT-3′, reverse primer: 5′-TCCACCACCCTGTTGCTGTA-3′). Each RT-qPCR reaction contained 10 *μ*l SYBR Premix Ex Taq (2x), 0.4 *μ*l forward and reverse primer, 1 *μ*l cDNA, 0.4 *μ*l Rox Reference Dye II, and dH_2_O up to the final volume of 20 *µ*L. PCR amplification was performed on a 7500 FAST Real-Time PCR System (Applied Biosystems) under the following cycling conditions: 30 s at 95°C, followed by 40 cycles at 95°C for 5 s and 60°C for 34 s. Each reaction was performed in triplicate. The 2^−ΔΔCt^ method was used to determine gene expression level [38].

## 3. Results

### 3.1. lncRNA Identification in Pigs

High-throughput transcriptome sequencing was performed to identify putative lncRNAs in a pool of samples from various pig tissues by strand-specific RNA sequencing. A total of 118 million high-quality paired-end 90 nt reads were obtained after eliminating adaptor sequences, eliminating low-quality reads, and filtering ribosomal RNA, of which 80.4% were successfully mapped to the pig reference genome. A total of 457,050 transcripts from 436,343 loci were assembled by Cufflinks [30]. Of the assembled sequences, 415,923 transcripts originated from single exons, whereas 41,127 transcripts contained multiexonic elements. Subsequently, this set of assembled sequences was used for lncRNA identification. By integrating information gained from our previous studies [22, 23], a pipeline including 7 stringent filtering steps was developed to identify putative* Sus scrofa* lncRNAs. We removed single-exon, short, and annotated transcripts, as well as those having coding potential. Finally, we identified a set of 2,139 lncRNAs located at 1,928 loci for further analysis (see Figure 1 and see Table S1 in Supplementary Material available online at https://doi.org/10.1155/2017/6152582). Moreover, we further evaluated the coding potential of putative lncRNAs by CPAT software; the results indicated 98.9% of the putative lncRNAs (2,115/2,139) were noncoding, indicating the high confidence of the lncRNAs we identified.

### 3.2. Sequence Characteristics of* Sus scrofa* lncRNAs

**Figure 2:**
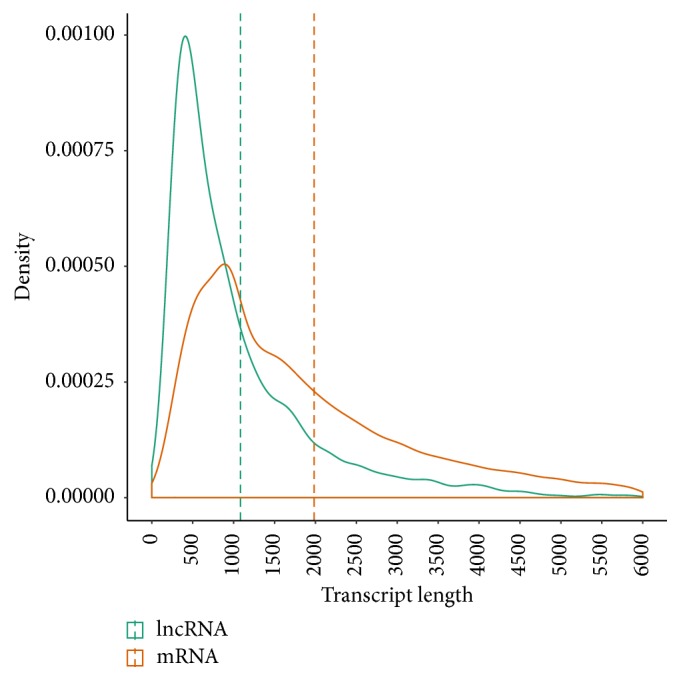
Transcript lengths of lncRNAs and protein-coding genes. The mean values (green and orange dashed line) of the transcript lengths are indicated.

**Figure 3:**
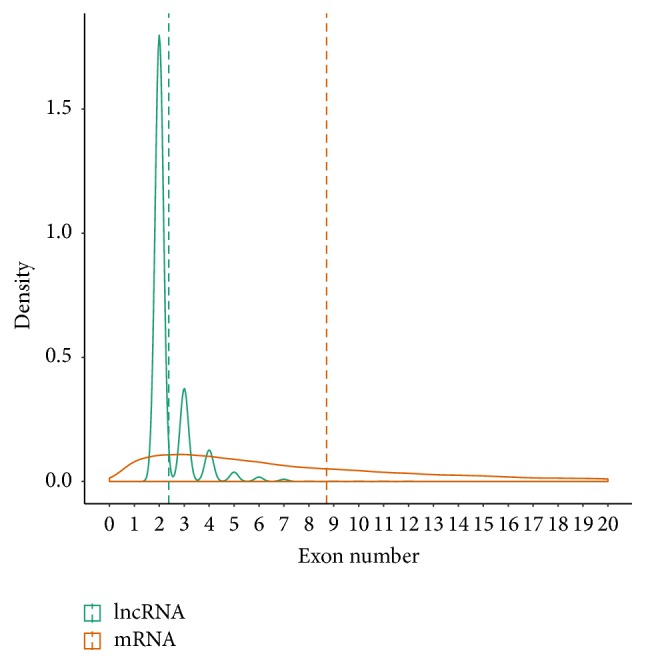
Exon numbers of lncRNAs and protein-coding genes. The mean values (green and orange dashed line) of the exon numbers are indicated.

To determine the features of* Sus scrofa* lncRNAs, we analyzed the sequence characteristics and expression levels of the lncRNAs and protein-coding genes (PCG) identified in the analysis described above. As shown in Figure 2, the average length of the lncRNAs was significantly shorter than that of the PCGs (1,082.7 nt versus 1,982.9 nt for lncRNAs and PCGs, resp.; Mann-Whitney* U* test, *P* < 2.2*e* − 16). Moreover, the lncRNAs also had fewer exons (mean number of exons, 2.38) than did the PCGs (mean number of exons, 8.71) (Mann-Whitney* U* test, *P* < 2.2*e* − 16) (Figure 3). FPKM (fragments per kilobase of exon per million fragments mapped) was chosen as a relative expression metric for the comparison of the expression levels of the lncRNAs with those of the PCGs. The expression levels of the lncRNAs were significantly lower than those of the PCGs (mean FPKM values, 1.93 versus 10.4 for lncRNAs and PCGs, resp.; *P* < 2.2*e* − 16). These results are consistent with those of previous studies of the expression levels of lncRNAs and PCGs in other mammals [22, 23, 39, 40].

### 3.3. GC Content of* Sus scrofa* lncRNAs

**Figure 4:**
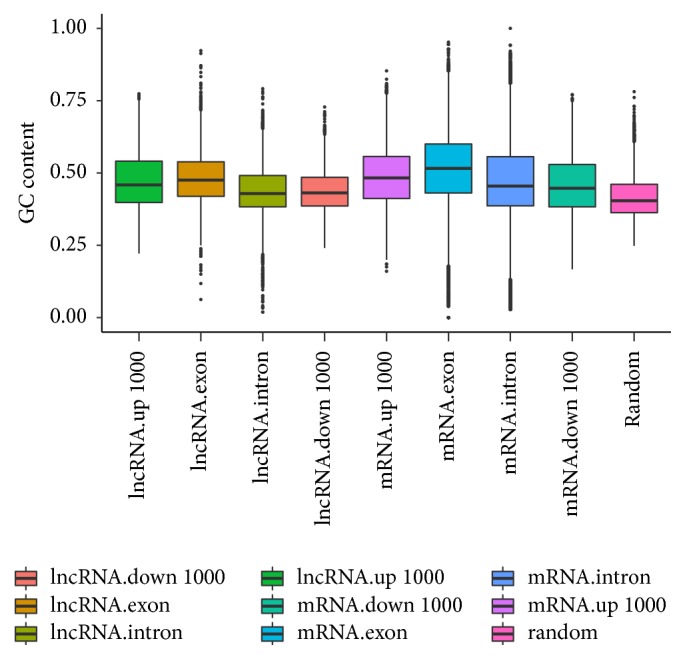
GC content of lncRNAs and protein-coding genes. The GC content of exons, introns, 1000 bp upstream regions, and 1000 bp downstream regions of lncRNAs and protein-coding genes, as well as that of random genome regions, is plotted as a box plot.

The GC content of exons, introns, and flanking regions (1000 bp upstream and 1000 bp downstream) of lncRNAs and PCGs, as well as that of 5,000 random genomic regions, was calculated, and differences in nucleotide composition were determined. As shown in Figure 4, the GC content of the lncRNA exons (48.15%) was greater than that of their introns (43.67%) (Mann-Whitney* U* test, *P* < 2.2*e* − 16) and flanking regions. The GC content of the regions upstream of lncRNAs (47.38%) was higher than that of their downstream regions (43.82%) (Mann-Whitney* U* test, *P* < 2.2*e* − 16). The GC content of the exons, introns, and flanking regions of PCGs showed differences similar to those observed for the corresponding regions of lncRNAs. The GC content of PCG exons (51.59%) was significantly higher than that of lncRNA exons and random genome regions (41.73%) (Mann-Whitney* U* test, *P* < 2.2*e* − 16). Moreover, the GC content of lncRNA exons was significantly higher than that of random genome regions (Mann-Whitney* U* test, *P* < 2.2*e* − 16).

### 3.4. SNP Density of* Sus scrofa *lncRNAs

**Figure 5:**
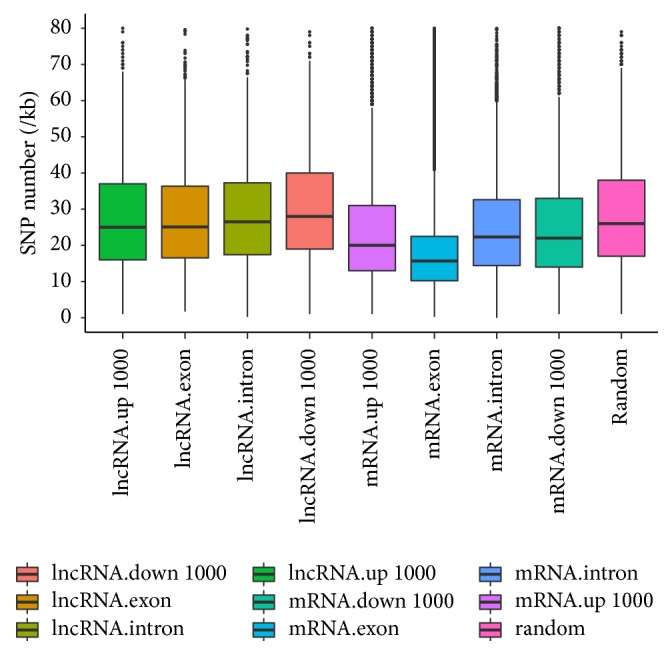
SNP density of lncRNAs and protein-coding genes. The SNP density of exons, introns, 1000 bp upstream regions, and 1000 bp downstream regions of lncRNAs and protein-coding genes, as well as that of random genome regions, is plotted as a box plot.

We also compared the nucleotide diversity of lncRNAs, PCGs, and random regions. SNP density (number of SNPs per unit physical length) was chosen as an indirect metric for the degree of sequence conservation. SNP density was calculated for lncRNAs, PCGs, and random genome regions. The SNP density of PCG exons (19.00 SNPs/kb), introns (24.59 SNPs/kb), and flanking regions (23.98 SNPs/kb for upstream regions and 25.42 SNPs/kb for downstream regions) was much lower than that of lncRNA exons (28.06 SNPs/kb), lncRNA introns (29.22 SNPs/kb), lncRNA flanking regions (upstream regions, 27.96 SNPs/kb; downstream regions, 30.56 SNPs/kb), and random regions (28.45 SNPs/kb) (Figure 5). These results reveal that, in* Sus scrofa*, the degree of sequence conservation of PGCs is greater than that of lncRNAs and other genome regions. However, no distinguishable difference in the SNP density of lncRNAs and random regions was observed (Mann-Whitney* U* test, *P* = 0.61). The SNP density of different parts of lncRNAs, PGCs, and mRNAs was also analyzed. The SNP density of exon regions was lower than that of intron regions in PGCs (*P* < 2.2*e* − 16) and lncRNAs (*P* = 0.035), which is consistent with reports that exons are conserved to a greater degree than are introns in many species.

### 3.5. Conservation Analysis of lncRNAs Across Humans, Mice, and Pigs

**Figure 6:**
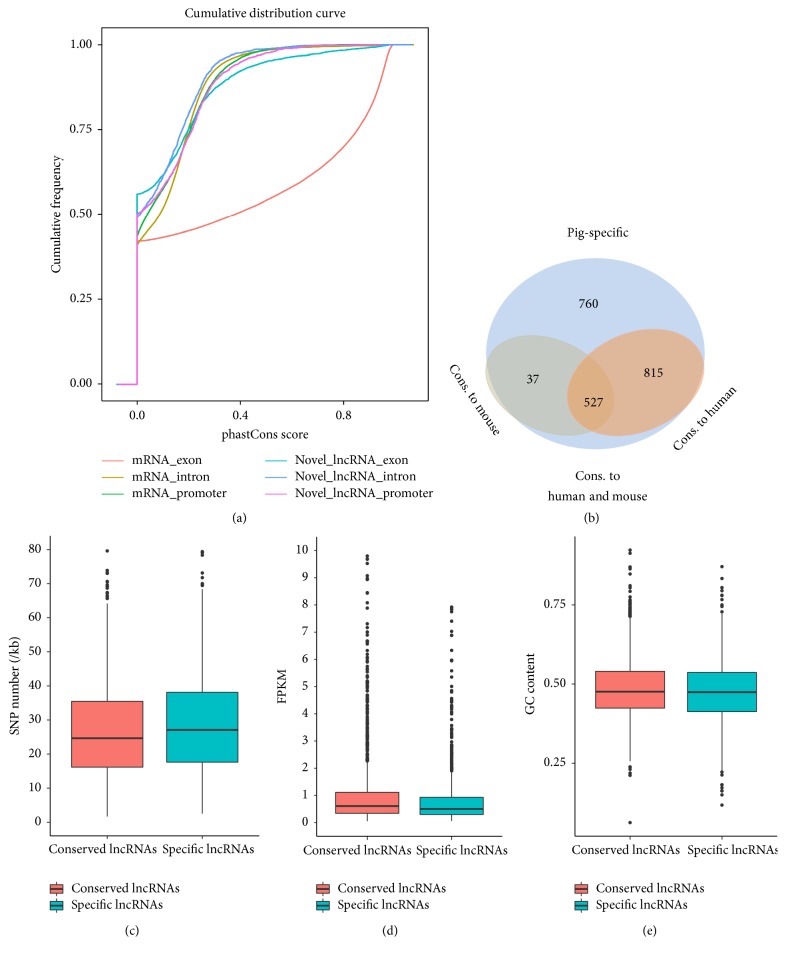
Conservation analysis of pig lncRNAs across species. (a) Cumulative curve of the average conservation score of the exons, introns, and promoter of protein-coding genes and predicted lncRNAs. The conservation was evaluated by phastCons scores. (b) The numbers of pig lncRNAs that can be aligned to multiple species are shown in the Venn diagram. (c) Box plots showing the SNP density of conserved and pig-specific lncRNAs. (d) Box plots showing the expression level of conserved and pig-specific lncRNAs. (e) Box plots showing the GC content of conserved and pig-specific lncRNAs.

**Figure 7:**
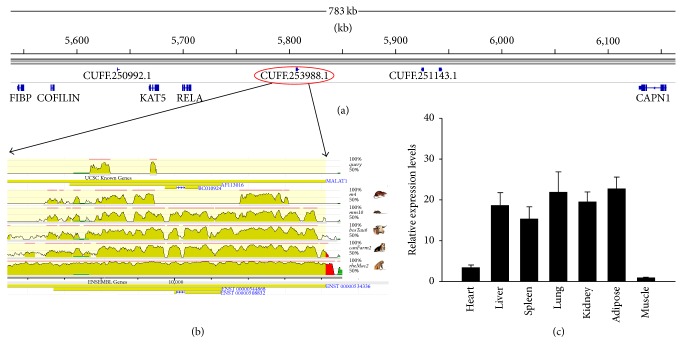
CUFF.253988.1 shares homology with human long noncoding RNA* MALAT1*. (a) The genome location of CUFF.253988.1 in pigs. (b) The graph shows the evolutionarily conservation of CUFF.253988.1 in different species, compared to the human genome (adapted from ECR browser, https://ecrbrowser.dcode.org/). The pig CUFF.253988.1 transcript is shown in the first line. (c) Expression pattern of CUFF.253988.1 in different pig tissues. The values are the mean (±SE) levels of CUFF.253988.1 from three independent experiments normalized to* GAPDH*.

We assessed the degree of evolutionary conservation of pig lncRNAs in the human and mouse genomes, because mice and pigs are both widely used as biomedical models for studies of human diseases. First, we compared the conservation degree between predicted lncRNAs and protein-coding transcripts by phastCons and observed that the exons of predicted lncRNAs were more conserved than the introns and promoters of lncRNAs. However, the exons of predicted lncRNAs were much less conserved than mRNA exons (Figure 6(a)). Next, the predicted lncRNAs in the analysis described above were classified into four groups (Figure 6(b)). The analysis of species conservation showed that 815 lncRNAs (38.1%) were conserved only in humans and pigs; 37 lncRNAs (1.7%) were conserved only in pigs and mice; 527 lncRNAs (24.6%) were conserved in humans, pigs, and mice; and 760 lncRNAs (35.5%) were not conserved in humans or mice and were therefore designated as pig-specific lncRNAs (Table S1). These results reveal that the sequences of most lncRNAs expressed by* Sus scrofa* are conserved in humans and mice. We then compared the characteristics differences between pig-specific lncRNAs and conserved lncRNAs. We found the SNP density of pig-specific lncRNAs (30.23 SNPs/kb) was significantly higher than that of conserved lncRNAs (27.06 SNPs/kb) (Mann-Whitney* U* test, *P* < 2.2*e* − 16) (Figure 6(c)), but the expression level and GC content of pig-specific lncRNAs were significantly lower than that of conserved lncRNAs (mean FPKM value, 1.49 vrsus 2.17, *P* = 4.6*e* − 06; mean GC content, 47.5 versus 48.4, *P* = 0.038, for lncRNAs and PCGs, resp.) (Figures 6(d) and 6(e)). Moreover, we detected homology of the putative lncRNAs with lncRNAs in human and mouse at transcript-level and found that 1299 (60.7%) and 860 (40.2%) of our lncRNAs can be aligned to human and mouse lncRNAs, respectively. For example, lncRNA-CUFF.253988.1, which is in the downstream of RELA protooncogene, NF-KB subunit* (RELA)* gene in pig genome (Figure 7(a)), shares homology with human long noncoding RNA metastasis associated lung adenocarcinoma transcript 1* (MALAT1)* and is evolutionarily conserved across species (Figure 7(b)). The expression profile analysis showed that CUFF.253988.1 gene was highly expressed in the adipose, lung, liver, kidney, and spleen and weakly expressed in the heart and* longissimus dorsi* (Figure 7(c)).

## 4. Discussion

The pig is a major agricultural animal and an important biomedical model, so it is necessary to understand the molecular regulatory mechanisms involved in their economic traits and the effects of diseases to which they are susceptible. The pig genome encodes a vast range of non-protein-coding RNAs (ncRNAs); however, information regarding pig lncRNAs is quite limited. For example, until this study, only 47 lncRNAs were deposited in the annotation file of the pig reference genome (v10.2) in the Ensembl database (release 78) [41]. The functions of most noncoding RNAs in pigs are unknown. Therefore, identification of pig lncRNAs and analysis of their characteristics are the first steps toward providing a foundation of knowledge regarding noncoding RNAs that will allow studies aimed at understanding their regulatory functions in pigs.

Using strand-specific total RNA sequencing, we developed a stringent pipeline to identify lncRNAs by integrating a set of previous approaches [22, 23], which allowed us to identify 2,139 high-confidence lncRNAs with strand information from an RNA pool. Seven steps were used to filter the high-confidence lncRNAs. Single-exon lncRNAs were filtered out to avoid transcriptional noise owing to the complexity of transcriptional reconstruction; this strategy was explored in several other studies [19, 39, 42, 43]. Moreover, four programs, CPC [31], CNCI [32], Pfam [44], and BLAST, were used to evaluate the coding potential of lncRNAs based on their sequence characteristics and protein databases. Moreover, the noncoding potential of putative lncRNAs was further assessed and confirmed by CPAT program. This approach significantly reduces the number of false positive results and ensures that lncRNAs were identified with high confidence. In comparison with known protein-coding transcripts, the putative lncRNAs have fewer exons, are shorter in transcript length, and have lower expression levels; these results are consistent with previous studies [22, 23].

GC content affects the structural stability of DNA/RNA and expression measurements for genomic features [45]. GC content varies substantially across the genome. For instance, the GC content of coding regions is usually greater than that of noncoding regions and the genome as a whole [46]. In this study, we confirmed that the GC content of protein-coding exons and introns was significantly greater than that of lncRNAs. The GC content of the exons of protein-coding genes and lncRNAs was greater than that of the introns of protein-coding genes and lncRNAs. Moreover, we found significant differences in the GC content of upstream regions, downstream regions, and random regions.

SNPs are frequently used as genetic markers to estimate genetic variation, evolutionary conservation, and natural selection [47]. We found that the SNP density of lncRNAs was significantly higher than that of protein-coding genes, perhaps because of the lesser degree of evolutionarily conservation of lncRNAs at the sequence level. Moreover, the SNP density of exon regions was lower than that of intron regions for both protein-coding genes and lncRNAs, which revealed that exons were conserved to a greater degree than were other genomic features.

Although lncRNAs have a generally lower level of sequence conservation and higher evolutionary rate in comparison with those of protein-coding genes, some lncRNAs are evolutionarily conserved across species [23]. Our study found that 24.6% of pig lncRNAs were conserved in the human and mouse genomes, providing new insight into the evolution of lncRNAs across species. As excepted, we observed the pig-specific lncRNAs were significantly higher conserved and lower expressed than conserved lncRNAs, which confirmed the higher conservation of conserved lncRNAs than pig-specific lncRNAs.

*MALAT1* was associated with a broad range of biological processes, such as cell cycle progression [48], alternative splicing [49], proliferation [50], and cell motility [51]. However, the function of* MALAT1* in pigs has not been reported. Interestingly, we found lncRNA-CUFF.253988.1, a homologous of* MALAT1*, was highly conserved across mammals and widely expressed in most tissues, implying that this lncRNA might play a wide range of roles in different tissues. However, further studies are needed to decipher the biological functions of* MALAT1* in pigs.

In summary, our genome-wide analysis achieved high-confidence identification and initial characterization of 2,139 lncRNAs in the pig genome using strand-specific RNA-seq technologies. The putative lncRNAs identified in this study provide a foundation for future studies of the biological functions of ncRNAs in* Sus scrofa*.

## Supplementary Material

Table S1. Summary of the predicted lncRNAs in *Sus Scrofa*.
